# Tamoxifen enhances the anticancer effect of cantharidin and norcantharidin in pancreatic cancer cell lines through inhibition of the protein kinase C signaling pathway

**DOI:** 10.3892/ol.2014.2711

**Published:** 2014-11-19

**Authors:** XIN XIE, MENG-YAO WU, LIU-MEI SHOU, LONG-PEI CHEN, FEI-RAN GONG, KAI CHEN, DAO-MING LI, WEI-MING DUAN, YU-FENG XIE, YI-XIANG MAO, WEI LI, MIN TAO

**Affiliations:** 1 Department of Oncology, The First Affiliated Hospital of Soochow University, Suzhou, Jiangsu 215006, P.R. China; 2 Department of Radiation Oncology, Affiliated Hospital of Xuzhou Medical College, Xuzhou, Jiangsu 221006, P.R. China; 3 Department of Hematology, The First Affiliated Hospital of Soochow University, Suzhou, Jiangsu 215006, P.R. China; 4 Jiangsu Institute of Hematology, The First Affiliated Hospital of Soochow University, Suzhou, Jiangsu 215006, P.R. China; 5 Key Laboratory of Thrombosis and Hemostasis of Ministry of Health, The First Affiliated Hospital of Soochow University, Suzhou, Jiangsu 215006, P.R. China; 6 Jiangsu Institute of Clinical Immunology, The First Affiliated Hospital of Soochow University, Suzhou, Jiangsu 215006, P.R. China

**Keywords:** pancreactic cancer, cantharidin, protein phosphatase 2A, tamoxifen, protein kinase C

## Abstract

Cantharidin is an active constituent of mylabris, a traditional Chinese therapeutic agent. Cantharidin is a potent and selective inhibitor of protein phosphatase 2A (PP2A). Cantharidin has been previously reported to efficiently repress the growth of pancreatic cancer cells. However, excessively activated protein kinase C (PKC) has been shown to improve cell survival following the adminstration of cantharidin. Tamoxifen is widely used in the treatment of estrogen receptor-positive breast cancer. In addition, an increasing number of studies have found that tamoxifen selectively inhibits PKC and represses growth in estrogen receptor-negative cancer cells. Administration of a combination of PKC inhibitor and PP2A inhibitors has been demonstrated to exert a synergistic anticancer effect. The proliferation of pancreatic cancer cells was analyzed by 3-(4,5-dimethyltiazol-2-yl]2, 5-diphenyltetrazo-lium bromide assay. The expression levels of ERα and ERβ in various pancreatic cancer cell lines were determined by reverse transcription polymerase chain reaction. In addition, the protein levels of PKCα and phosphorylated PKCα in pancreatic cell lines were analyzed by western blot analysis. In the present study, tamoxifen was found to exert a cytotoxic effect against pancreatic cancer cells independent of the hormone receptor status. Tamoxifen repressed the phosphorylation of PKC, and amplified the anticancer effect induced by cantharidin and norcantharidin. The findings reveal a novel potential strategy against pancreatic cancer using co-treatment with tamoxifen plus cantharidin or cantharidin derivatives.

## Introduction

Pancreatic cancer is a solid malignancy with one of the highest current mortality rates. In spite of decades of effort, the five-year survival rate remains at only ~5%. No early detection tests have been developed, and the majority of patients with localized disease exhibit no identifiable signs or symptoms. Therefore, patients are often not diagnosed until the late stages of disease, when the cancer has metastasized to other organs (1). Fewer than 20% of patients are eligible for potentially curative resection. However the majority of these patients exhibit disease recurrence (2). Thus, effective adjuvant therapies are urgently required.

Numerous studies support the hypothesis that cantharidin and cantharidin derivatives exert marked *in vitro* and *in vivo* antitumor activity against various types of cancer cell (3–5). In previous studies, cantharidin was found to repress cancer cell growth through cell cycle arrest and the induction of apoptosis (6–9). Norcantharidin is a derivate of cantharidin, which is more widely used in clinical trials with less kidney toxicity. Cantharidin and norcantharidin act as potent and selective inhibitors of protein phosphatase 2A (PP2A), a multimeric serine/threonine phosphatase. Inhibition of PP2A is considered to promote cancer development through the induction of phosphorylation and activation of several substrate kinases, including IκB kinase, c-Jun N-terminal kinase (JNK), extracellular signal-related kinase, p38, Akt and protein kinase C (PKC), the majority of which accelerate growth (10,11). However, recent studies have shown that several kinase-dependent growth inhibition pathways are induced by treatment with PP2A inhibitors (12,13). We previously showed that cantharidin exerts an anticancer effect through overactivation of the JNK signaling pathway, while excessively activated PKC impaired the anticancer effect of cantharidin (8). The combination of PP2A inhibitors and PKC inhibitor was demonstrated to produce a synergistic effect against pancreatic cancer cells (8). However, the PKC inhibitor used in our previous study, GF109203X, has not been commonly used in clinic trials. Thus, a PKC inhibitor with demonstrated clinical safety may be more suitable for use in combination treatment with PP2A inhibitors in future clinical trials.

Tamoxifen is a synthetic nonsteroidal antiestrogen agent widely used for the endocrinotherapy of breast cancer. Notably, tamoxifen also inhibits the growth of estrogen receptor (ER)-negative cell lines (14,15). Previous studies have demonstrated that inhibition of PKC may be the underlying mechanism by which tamoxifen exerts antiproliferative effects against ER-negative cell lines (16–19). Thus, in the present study, tamoxifen-mediated inhibition of the PKC signaling pathway and cell proliferation in pancreatic cancer cells was investigated, together with the synergistic anticancer effect using the combination of tamoxifen plus cantharidin or norcantharidin.

## Materials and methods

### Cell lines and culture

MCF-7 and MDA-MB-231 breast cancer cell lines were purchased from the American Type Culture Collection (Manassas, VA, USA) and maintained in RPMI-1640 (Gibco-BRL, Grand Island, NY, USA) supplemented with 10% fetal calf serum (FCS; HyClone Laboratories, Inc., Logan, UT, USA), 100 U/ml penicillin and 100 mg/ml streptomycin at 37°C in a humidified atmosphere with 5% CO_2_. PANC-1, BxPC-3, CFPAC-1, Capan-1, PL-45 and SW-1990 human pancreatic cancer cell lines were purchased from the American Type Culture Collection and maintained in Dulbecco’s modified Eagle’s medium (Gibco-BRL) supplemented with 10% FCS (HyClone Laboratories, Inc., Logan, UT, USA), 100 U/ml penicillin and 100 mg/ml streptomycin at 37°C in a humidified atmosphere with 5% CO_2_. The cells were passaged every 2–3 days to maintain exponential growth.

### Reagents

Cantharidin, tamoxifen and GF109203X were purchased from Enzo Life Science International, Inc. (Plymouth Meeting, PA, USA). Norcantharidin was purchased from Sigma-Aldrich (St. Louis, MO, USA).

### 3-[4,5-dimethyltiazol-2-yl] 2,5-diphenyl-tetrazolium bromide (MTT) assay

Cellular viability and growth was evaluated by MTT assay (20). The cells were seeded into 24-well tissue culture plates at 5×10^4^ cells/well. Subsequent to treatment, MTT (Sigma-Aldrich) at a final concentration of 0.5 mg/ml was added to each well and the cells were incubated at 37°C for 4 h. The medium was then removed and 800 μl dimethyl sulfoxide was added to each well. The absorbance of the mixture was measured at 490 nm using a microplate ELISA reader (Model 680; Bio-Rad, Hercules, CA, USA*)*. The relative cell viability was calculated as follows: Relative cell viability = (mean experimental absorbance/mean control absorbance) × 100%. The growth inhibition rate was calculated as follows: Inhibition rate = [(mean control absorbance − mean experimental absorbance)/mean control absorbance] × 100%.

### Reverse transcription-polymerase chain reaction (RT-PCR)

RT-PCR was performed to estimate the expression levels of ER mRNA. In brief, total RNA was extracted using TRIzol reagent (Invitrogen Life Technologies, Carlsbad, CA, USA) according to the manufacturer’s instructions. Following spectrophotometric quantification, 1 μg total RNA in a final volume of 20 μl was used for reverse transcription using Avian Myeloblastosis Virus reverse transcriptase (Promega, Madison, WI, USA) according to the manufacturer’s instructions. The RT-PCR reaction products were electrophoresed on 1.5% agarose gels, visualized using ethidium bromide staining and quantified using Quantity One software (Bio-Rad). β-actin served as the internal positive control and as the reference gene for PCR cycle number normalization. This ensured linear amplification of the templates in each experiment. The primers used in PCR were as follows: Forward, 5′-AGGGTAAATGGTAGTTGAAAGGA-3′ and reverse, 5′-ACGCTGGGAAATGAAGAAGA-3′ for ER-1 (product, 280 bp); forward, 5′-TTTAGTGGTCCATCGCCAGTTA-3′ and reverse, 5′-CAGCTCTTGCGCCGGTTT-3′ for ER-2 (product, 339 bp); and forward, 5′-TCATGAAGTGTGACGTGGACAT-3′ and reverse, 5′-CTCAGGAGGAGCAATGATCTTG-3′ for β-actin (product, 158 bp).

### Western blot analysis

Monoclonal mouse anti-PKCα and mouse anti-human β-actin antibodies were purchased from Santa Cruz Biotechnologies (Santa Cruz, CA, USA), and polyclonal rabbit anti-human phospho-PKCα (Thr638) antibodies were purchased from Cell Signaling Technology, Inc. (Beverly, MA, USA). Total protein was extracted using a lysis buffer containing 50 mm Tris-HCl (pH 7.4), 150 mm NaCl, 1% Triton X-100, 0.1% SDS and 1 mm EDTA, supplemented with protease inhibitor cocktail (Roche, Indianapolis, IN, USA) and phosphatase inhibitor cocktail (Roche). The protein extract was loaded onto an SDS-polyacrylamide gel, size-fractionated by electrophoresis and then transferred to polyvinylidene fluoride membranes (Bio-Rad Laboratories). Subsequent to blocking in 5% non-fat milk for 1 h, the membranes were incubated overnight with the primary antibodies at 4°C. Protein expression was determined using horseradish peroxidase-conjugated secondary monoclonal goat anti-rabbit IgG and goat anti-mouse IgG antibodies (sc-2004 and sc-2005, respectively; Santa Cruz Biotechnologies) followed by enhanced chemiluminescence detection (Amersham Pharmacia Biotech, Amersham, UK). β-actin served as the internal control. Analysis of grays was performed using Quantity One 4.6.2 software (Bio-Rad).

### Statistical analysis

Each experiment was performed a minimum of three times. The results are expressed as the mean± standard deviation. Statistical analysis was performed using an unpaired Student’s t-test. P<0.05 was considered to indicate a statistically significant difference.

## Results

### Tamoxifen represses growth of pancreatic cancer cells in a hormone receptor-independent manner

**Figure 1:**
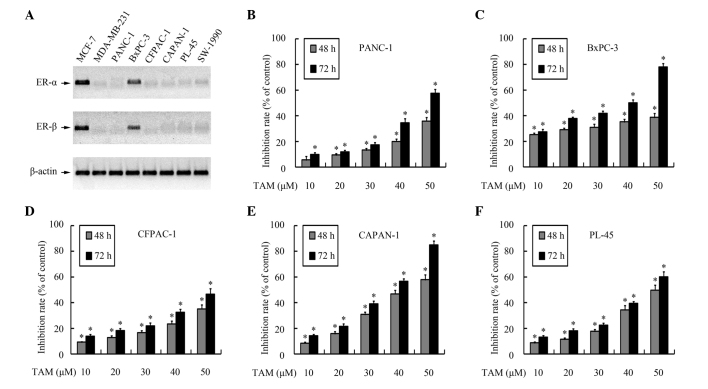
Hormone receptor status in pancreatic cancer cell lines and inhibitory effect of tamoxifen (TAM) on the growth of pancreatic cancer cells. (A) Reverse transcription-polymerase chain reaction experiments for detecting hormone receptor expression. MCF-7 and MDA-MB-231 breast cancer cells served as estrogen receptor (ER)-positive and -negative controls respectively. (B–F) Exposure to various concentrations of tamoxifen resulted in dose- and time-dependent growth inhibition of (B) PANC-1, (C) BxPC-3, (D) CFPAC-1, (E) CAPAN-1 and (F) PL-45 pancreatic cancer cells. ^*^P<0.05 and ^**^P<0.01, as compared with the respective control groups for each time point.

As hormone receptors are the main target of tamoxifen, the expression of ER-α and ER-β in the pancreatic cancer cell lines was evaluated using RT-PCR. As shown in Fig. 1A, hormone receptor expression was detected in the BxPC-3 cells, but the other pancreatic cell lines were observed to be hormone receptor-negative.

The effects of tamoxifen on the growth of pancreatic cancer cells were then analyzed using MTT assays. As shown in Fig. 1B–F, tamoxifen inhibited cell growth in a dose- and time-dependent manner, not only in the hormone receptor-positive cell line, but also in the hormone receptor-negative cells lines, which suggests that tamoxifen repressed growth in pancreatic cancer cells independently of the hormone receptor status.

### Tamoxifen inhibits proliferation of pancreatic cancer cells through PKC suppression

PKC inhibition has been previously shown to be the predominant mechanism involved in the ER-independent anticancer effect of tamoxifen (21). As tamoxifen-mediated cytotoxicity was demonstrated in ER-positive and -negative pancreatic cancer cells, whether this hormone receptor-independent inhibition effect was mediated through inhibition of PKC was then investigated.

**Figure 2:**
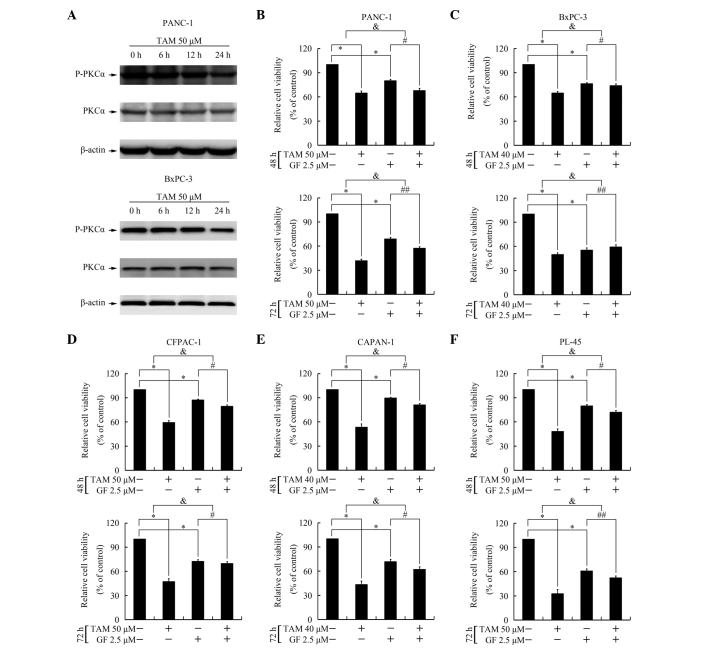
Tamoxifen inhibits pancreatic cancer cell proliferation in a protein kinase C (PKC) pathway-dependent manner. (A) Treatment with tamoxifen (TAM) induced time-dependent downregulation of PKCα phosphorylation in PANC-1 and BxPC-3 cells. (B–F) The time-dependent cytotoxic effect of tamoxifen was repressed by GF109203X (GF) in (B) PANC-1, (C) BxPC-3, (D) CFPAC-1, (E) CAPAN-1 and (F) PL-45 pancreatic cancer cells. ^*^P<0.05, vs. the respective control groups; ^#^P<0.05 and ^##^P<0.01, vs. the GF109203X group; ^&^P<0.05 and ^&&^P<0.01, fold change following treatment.

PKC repression by tamoxifen was confirmed using western blotting. As shown in Fig. 2A, treatment with tamoxifen reduced PKCα phosphorylation. The time-dependent repression of cell viability induced by tamoxifen was attenuated by pretreatment with GF109203X, an inhibitor of PKC (Fig. 2B–F), which suggests that tamoxifen repressed pancreatic cancer cell viability in a PKC pathway-dependent manner.

### PP2A inhibitors suppress the growth of pancreatic cancer cells

**Figure 3:**
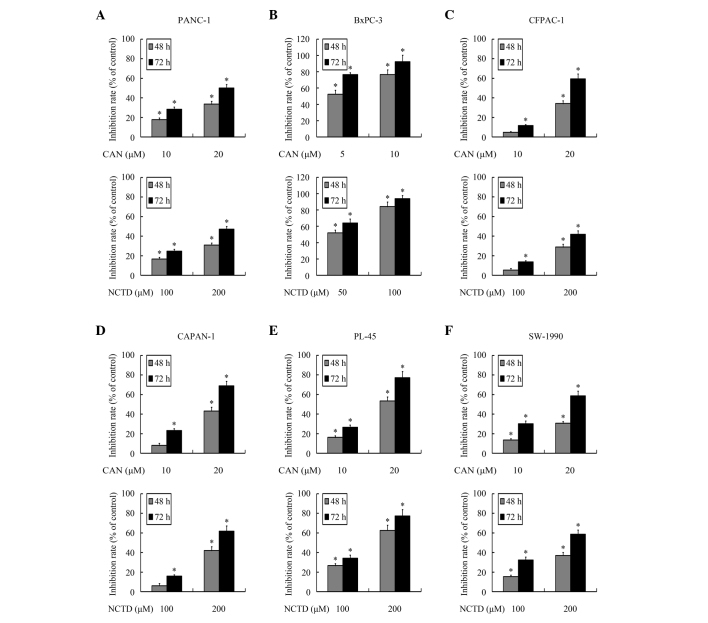
Exposure to various concentrations of cantharidin (CAN) and norcantharidin (NCTD) results in dose- and time-dependent growth inhibition in (A) PANC-1, (B) BxPC-3, (C) CFPAC-1, (D) CAPAN-1, (E) PL-45 and (F) SW-1990 pancreatic cancer cells. ^*^P<0.05 and^**^P<0.01, as compared with the respective control groups for each time point.

Cantharidin has been previously reported to repress the growth of pancreatic cancer cell lines (6). However, whether norcantharidin also exerts a comparable cytotoxicity effect against pancreatic cancer cells remains unknown. To investigate the cytotoxic effect of norcantharidin, MTT assays were performed. As presented in Fig. 3, both cantharidin and norcantharidin treatment markedly repressed the growth of PANC-1, BxPC-3, CFPAC-1, CAPAN-1, PL-45 and SW-1990 cells in a dose- and time-dependent manner.

### Tamoxifen represses the PKC phosphorylation induced by PP2A inhibitors and increases the cytotoxicity mediated by PP2A inhibitors

**Figure 4:**
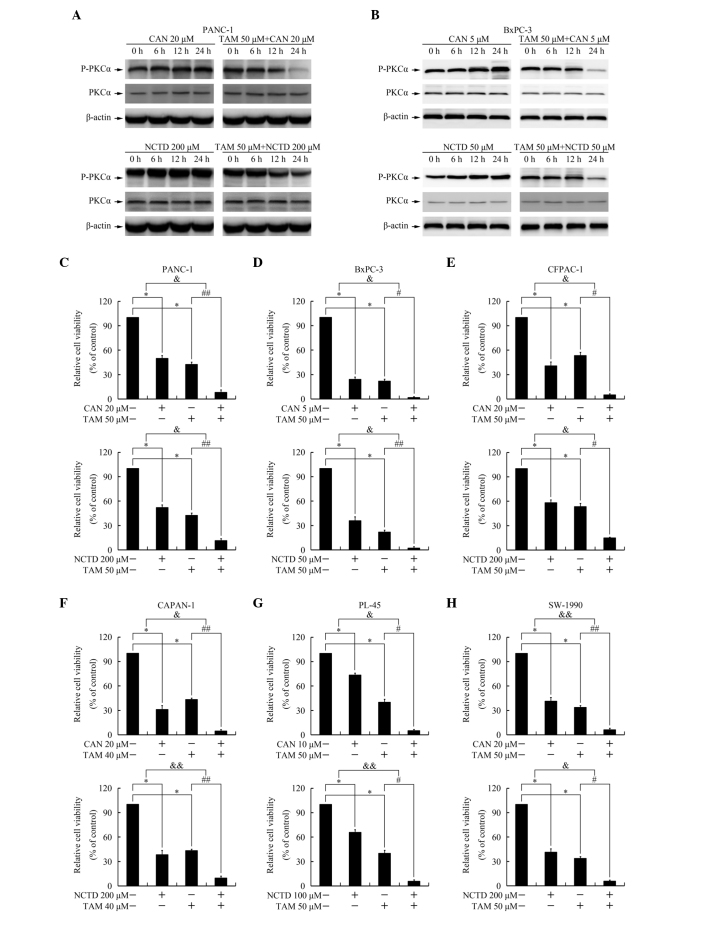
Tamoxifen increases cantharidin and norcantharidin cytotoxicity through inhibition of the protein kinase C (PKC) signaling pathway. (A and B) Treatment with cantharidin (CAN) or norcantharidin (NCTD) induced PKCα phosphorylation, but PKCα expression did not exhibit significant changes. Tamoxifen (TAM) repressed the PKCα phosphorylation stimulated by cantharidin and norcantharidin. (C–H) The cytotoxic effect of cantharidin or norcantharidin was elevated by tamoxifen in (C) PANC-1, (D) BxPC-3, (E) CFPAC-1, (F) CAPAN-1, (G) PL-45 and (H) SW-1990 pancreatic cancer cells. ^*^P<0.05, vs. the respective control groups; ^#^P<0.05 and ^##^P<0.01, vs. the tamoxifen group; ^&^P<0.05 and ^&&^P<0.01, fold change following treatment.

As shown in Fig. 4A and B, cantharidin and norcantharidin induced persistent and excessive phosphorylation of PKCα, an effect repressed by pretreatment with tamoxifen, which suggests that tamoxifen may act as a PKC inhibitor and inhibit the activation of PKC induced by the PP2A inhibitors. Administration of a combination of PKC inhibitors has previously been reported to increase cantharidin-mediated cytotoxicity (8). To investigate whether tamoxifen also exerts a similar effect, an MTT assay was performed. As shown in Fig. 4C–H, combination treatment with tamoxifen increased the cytotoxicity of cantharidin and norcantharidin, exerting a synergistic effect.

## Discussion

Exocrine pancreatic cancer is significantly more frequent in young males than in young females. The male-to-female ratio is 1.25–1.75:1, but is reduced with increasing age (22). ERs and estrogen-binding proteins are present in the human healthy pancreas, and experimental pancreatic cancer has been shown to be influenced by estrogens (22). These investigations have raised interest in sex hormones in the development of pancreatic cancer and in the application of endocrinotherapy in the treatment of pancreatic cancer (22).

Tamoxifen is a prototypical drug that targets the ER. Tamoxifen exerts potent antiestrogenic activity and has been used extensively for the past 40 years to treat and prevent breast cancer (23). Although tamoxifen administered alone has repeatedly been shown to not exert a significant effect against pancreatic cancer in clinical studies (22), combination therapy comprising tamoxifen with other chemotherapeutic agents has been shown to be effective in phase II trials, regardless of the hormone receptor status of the pancreatic tumor (24,25). These studies suggest that tamoxifen may be able to increase the cytotoxicity of other agents against pancreatic cancer in a hormone receptor-independent manner.

The inhibition of ER-negative breast cancer cells and other cell types by tamoxifen has been previously investigated. Studies have hypothesized that this off-target effect of tamoxifen involves PKC inhibition (18). The present study found that tamoxifen treatment repressed the growth of pancreatic cancer cell lines, independent of the hormone receptor status. This tamoxifen-mediated cytotoxicity was attenuated by the PKC inhibitor, GF109203X. Thus, the growth-inhibitory activity of tamoxifen on pancreatic cancer cells may partially be due to PKC inhibition. Western blot analyses revealed that tamoxifen significantly repressed the phosphorylation of PKCα stimulated by cantharidin and norcantharidin. Furthermore, co-treatment with tamoxifen increased cantharidin- and norcantharidin-mediated cytotoxicity, exerting a synergistic effect. Thus, tamoxifen, as a PKC inhibitor widely used in clinical practice, may increase the cytotoxic effect of cantharidin and norcantharidin treatment, regardless of the hormone receptor status.

Multi-component therapy, termed herbal formulae in traditional Chinese medicine, in which two or more agents interact with multiple targets simultaneously, is considered as a rational and efficient medicinal system designed to treat various illnesses, including cancer (26,27). As determined by the symptoms and characteristics of patients, herbal formulae are designed to contain a combination of different types of plants or minerals in the order of ‘Jun (monarch)-Chen (assistant)-Zuo (minister)-Shi (guide)’ (28). In a traditional formula, the ‘Jun’ (monarch) drug is an required ingredient in a prescription, and exerts a leading curative role aimed at the cause or the predominant syndrome of a disease. The ‘Chen’ (minister) drug strengthens the curative effect of the ‘Jun’ drug or treats the accompanying symptoms, if applicable. The ‘Zuo’ (assistant) drug mainly coordinates the formula, increasing the therapeutic effects of ‘Jun’ and ‘Chen’ and reducing the side-effects. The ‘Shi’ (guide) drug directs the other drugs in the prescription to the affected area or regulates the properties of the other components. Over thousands of years, almost 100,000 formulae have been recorded by practitioners according to experience and heritage from ancestors, but the mechanisms involved in the majority of these formulae remain unclear. With the development of molecular biology techniques, an increasing number of composition principles of ‘Jun-Chen-Zuo-Shi’ Chinese compound prescriptions have been analyzed, examining the chemical components, precise mechanisms of action, clinical application and efficacy validation (29–32). For example, the Chinese medicinal formula Realgar-Indigo naturalis has been shown to be effective in treating promyelocytic leukemia, in which tetraarsenic tetrasulfide is the ‘Jun’ drug, and ndirubin and tanshinone IIA act as the ‘Chen’ drugs. The combination of these three drugs yields synergy in the treatment of a murine acute promyelocytic leukemia (29). These findings not only demonstrate the molecular mechanism of formula at the molecular biology level, but also indicate that formulae may exert therapeutic effects through mechanisms beyond each individual component.

Ginseng and *Astragalus membranaceus* are two Chinese medicinal herbs commonly used in herbal formulae that contain mylabris. In these formulae, mylabris is considered to be the ‘Jun’ drug, and Ginseng and *Astragalus membranaceus* are known as the ‘Chen’ drugs. Notably, studies have demonstrated that Ginsenosides, the predominant active constituent of ginseng, and Astragaloside, a saponin purified from *Astragalus membranaceus*, are capable of inhibiting PKC (33,34). Thus, the regulation of the PKC signaling pathway by these herbs may be involved in the synergistic mechanism of these formulae. However, Ginsenosides and Astragaloside are also able to repress the JNK signaling pathway (35,36), the activation of which is the main mechanism of cantharidin cytotoxicity (6,8). Repression of JNK by Ginsenosides and Astragaloside may impair the anticancer effect of cantharidin, which suggests that a relatively specific PKC inhibitor may be a more effective choice in a multi-component therapy.

In our previous study, the addition of a PKC inhibitor, GF109203X, to treatment regimes was found to increase the cytotoxicity of cantharidin (8). However, GF109203X has not been applied in clinical trials. Furthermore, cantharidin cytotoxicity to the normal hepatic and urinary system tissues restricts the clinical application of this drug (9). Thus combination treatment with PKC inhibitors and PP2A inhibitors requires practical candidate therapeutic agents. As the PKC inhibitor, tamoxifen, and the demethylated form of cantharidin, norcantharidin, have been shown to exhibit adequate efficacy, safety and compliance in the clinical setting, the combination of these agents may become a promising adjuvant therapy formula in the treatment of pancreatic cancer.
